# Physiological cyclic stretch up-regulates angiotensin-converting enzyme 2 expression to reduce proliferation and migration of vascular smooth muscle cells

**DOI:** 10.1042/BSR20192012

**Published:** 2020-06-15

**Authors:** Jiantao Song, Haiyan Qu, Bo Hu, Chenglong Bi, Mengmeng Li, Lin Wang, Xiaozhen Huang, Mei Zhang

**Affiliations:** 1 The Key Laboratory of Cardiovascular Remodeling and Function Research, Chinese Ministry of Education, Chinese National Health Commission and Chinese Academy of Medical Sciences, The State and Shandong Province Joint Key Laboratory of Translational Cardiovascular Medicine, Qilu Hospital of Shandong University, 107 Wenhuaxi Road, 250012 Jinan, China; 2 Department of Emergency, Shandong Provincial Hospital Affiliated to Shandong First Medical University, Jinan, China; 3 Department of Cardiology, The First Affiliated Hospital of Shandong First Medical University, Jinan, China

**Keywords:** ACE2, cyclic stretch, HASMCs, migration, proliferation

## Abstract

Angiotensin-converting enzyme 2 (ACE2) is considered as an endogenous negative regulator of renin–angiotensin system (RAS), exerting multiple cardiovascular protective roles. Whether mechanical stretch modulates ACE2 expression remains unknown. The present study aimed at investigating whether ACE2 is involved in physiological stretch (10% elongation, 1 Hz) mediated cellular functions and the underlying mechanism. Cultured human aortic smooth muscle cells (HASMCs) were exposed to 10% stretch for indicated time, and real-time PCR and Western blot analysis showed 10% stretch increased ACE2 expression and activity significantly compared with static conditions and increased Ang-(1-7) level, but decreased Ang II level; Brdu incorporation assay and Scratch test showed that ACE2 was involved in the inhibition of HASMCs proliferation and migration by 10% stretch; the Dual-Luciferase Reporter Assay demonstrated that 10% increased ACE2 promoter activity, but had no effect on ACE2 mRNA stability; kinase inhibition study and Electrophoretic mobility shift assay (EMSA) showed that JNK1/2 and PKCβII pathway, as well as their downstream transcription factors, AP-1 and NF-κB, were involved in 10% stretch induced ACE2 expression. In conclusion, our study indicates ACE2 is a mechanosensitive gene, and may represent a potential therapeutic target for mechanical forces related vascular diseases.

## Introduction

The blood vessels are constantly exposed to mechanical forces in the form of cyclic stretch and shear stress due to pulsatile blood pressure and blood flow, respectively. Shear stress mainly regulates functions of endothelial cells; however, stretch directly affects all cell types of vessel wall and vascular smooth muscle cells (VSMCs) are the main target cells [1]. A growing body of evidence suggest that stretch affects multiple cellular functions of VSMCs, including proliferation, migration, apoptosis, phenotypic switching, and extracellular matrix (ECM) remodeling [2]. Under physiological conditions, the human aorta undergoes approximately 10% circumferential stretch, which plays a critical role in maintaining vascular homeostasis [3], while increased stretch, as occurs in hypertension condition, can promote the dysregulation of VSMCs functions [4].

The renin–angiotensin system (RAS) plays important roles in regulating blood pressure and vascular homeostasis [5]. Overactivation of RAS is closely associated with various cardiovascular diseases, including hypertension and atherosclerosis [6]. Angiotensin II (Ang II) is the main effective peptide of RAS, exerting many adverse cardiovascular effects via binding its type I receptor (AT1R). Blockade of RAS with angiotensin-converting enzyme (ACE) inhibitors or AT1 receptor antagonists has been proven to be effective for many cardiovascular diseases [7,8]. ACE2, a homologue of ACE, degrades Ang II into Ang-(1-7), a peptide with cardiovascular protective effects as compared with the adverse effects of Ang II, functioning as an endogenous negative regulator of RAS [9,10]. Recently, several lines of evidence indicate that ACE2 is a potential therapeutic target for many cardiovascular diseases. Previous studies confirmed that Ang II and its receptor, AT1, were involved in stretch-mediated cellular functions [11–14]. However, the effect of stretch on ACE2 expression in VSMCs remains unclear. Because of the important roles of ACE2 in hypertension and vascular remodeling, we propose a hypothesis that ACE2 is also a mechanical stretch sensitive gene, which plays important roles in regulating cellular functions of VSMCs by stretch.

## Methods

### Materials

Antibodies against ACE2 and phosphorylated Sp1 (p-Sp1) were from Abcam (Cambridge, U.K.). Antibodies against total and/or phosphorylated c-jun, c-fos, p65, Sp-1, extracellular signal-regulated kinase 1/2 (ERK1/2), c-Jun kinase 1/2 (JNK1/2), p38, and protein kinase C βII (PKCβII) were from Cell Signaling Technology (Boston, MA). Lipofectamine 2000 was from Invitrogen (Carlsbad, CA). Pharmacological inhibitors for p38 MAPK (SB203580), PI3K/Akt (LY294002), ERK1/2 (PD98059), and JNK1/2 (SP600125) were from Cell Signaling Technology. CG53353 (PKCβII inhibitor) was from EMD Chemicals (Philadelphia, PA); Curcumin (AP-1 inhibitor) was from Sigma-Aldrich; SN50 (NF-κB inhibitor) and WP631 (Sp1 inhibitor) were from Alexis Biochemicals (Lausen, Switzerland).

### Cell culture and application of cyclic stretch

Human aortic smooth muscle cells (HASMCs), purchased from ScienCell (U.S.A.), were cultured in SMC medium (ScienCell, U.S.A.) with 5% CO_2_ at 37°C. Passages 4–7 cells were seeded (10^5^ cells/well) onto six-well Flexcell plates coated with collagen I, and when they reached 90% confluence, serum-free medium was added to induce quiescence for 24 h and replaced with fresh complete medium, then physiological stretch (10% elongation, 1 Hz) was applied by use of a computer-controlled Flexcell 5000-Tension apparatus (Flexercell Strain Unit, FlexCell International). The static control cells subjected to the same conditions as the experimental cells, except that they were not exposed to stretch. For inhibition of stretch-induced activation of transcription factors and signaling pathways, pharmacological inhibitors were added to the culture media 1 h before stretch treatment.

### Western blot analysis

After being stretched for the indicated time, cells were harvested and protein was extracted by use of a nuclear and cytoplasmic extraction reagent kit (Pierce, Thermo Scientific) containing protease inhibitor cocktail (Pierce). Protein extracts (20 μg) were separated by SDS-PAGE, transferred to PVDF membrane (Millipore, MIT, U.S.A.), which was blocked with 5% nonfat milk for 2 h, then incubated with primary antibodies against target proteins overnight at 4°C, then incubation with secondary antibodies (ZSGB-BIO, Beijing) at room temperature for 1 h. Target proteins bands were visualized by use of an enhanced chemiluminescence plus detection system (Millipore). Relative band intensities were analyzed by use of Photoshop CS3.

### RNA isolation and RT-PCR

The total RNA was extracted by use of TRIzol Reagent (Invitrogen) according to the of the manufacturer’s instructions, and converted to cDNA via the PrimeScript® RT reagent kit (Takara Biotechnology, Dalian, China), then underwent one-step real-time PCR with SYBR Green technology on a Light Cycler (Bio-Rad, Hercules, CA) to determine the mRNA level of ACE2, ACE, and MAS (the receptor of Ang-(1-7)). The primer sequences were designed as ACE2 (sense: 5′-CATTGGAGCAAGTGTTGGATCTT-3′, antisense: 5′-GAGCTAATGCATGCCATTCTCA-3′); ACE (sense: 5′-GCGGCTCTTCCAGGAGCTGC-3′, antisense: 5′-CTGCGCCCACATGTTCCCCA-3′); MAS (sense: 5′-GGCCTCCTCATGGATGGGTCAA-3′′, antisense:5′-GTGCATTCCCGACTGAGGCGT-3′); and β-actin (sense: 5′-TGACGTGGACATCCGCAAAG-3′, antisense: 5′- CTGGAAGGTGGACAGCGAGG -3′) as a normalization control. PCR products were electrophoresed through 1.5% agarose gels. The data were analyzed by the 2^−ΔΔCT^ method.

### ACE2 activity assay

ACE2 activity was determined by SELDI-TOF-MS as previously described (Liu et al, 2011). In brief, 20 μg of total protein extract was incubated with 7-Mca-YVADAPK (Dnp, 1 μM) (R&D Systems, U.S.A.), a substrate of ACE2, in a final volume of 100 μl reaction buffer at room temperature. Fluorescence kinetics was measured for 4 h by use of Varioskan Flash (Thermo Scientific, Worcester, MA, U.S.A.) at excitation 320 nm and emission 400 nm. EDTA (1 mM) and recombinant human ACE2 (25 ng, R&D Systems, U.S.A.) were used as a positive control. ACE2 activity was expressed as the difference in fluorescence with or without the ACE2 inhibitor DX600 (1 μM, Belmont, CA, U.S.A.).

### ELISA measurement of Ang II and Ang-(1-7) levels

Cytoplasmic protein was extracted from HASMCs by use of a commercial kit (Pierce), and protein concentration was determined by BCA assay. Cytoplasmic protein from each well was stored at −80°C. Commercial ELISA kits were used to evaluate levels of Ang II (SPI-BIO, France) and Ang-(1-7) (Bachem, U.S.A.).

### RNA interference

Small interfering RNA (siRNA) for ACE2 and scramble control siRNA were synthesized by GenePharma (Shanghai, China). The sequences for ACE2 siRNA were (sense: 5′-CCA UCU ACA GUA CUG GAA A dTdT-3′, antisense, 5′-UUU CCA GUA CUG UAG AUG G dTdT-3′); for scramble control siRNA, (sense, 5′-UUC UCC GAA CGU GUC ACG U dtdt-3′, antisense, 5′-ACG UGA CAC GUU CGG AGA A dtdt-3′). For siRNA transfection, HASMCs at 70–80% confluence were transfected with ACE2 siRNA or control siRNA at 30 nM for 48 h, and then subjected to the next experiments.

### Cell proliferation analysis

Cell proliferation analysis involved cell counting and BrdU incorporation. HASMCs were exposed to physiological stretch (10% elongation, 1 Hz) for 12 h, and the medium was removed and washed with cold phosphate-buffered saline (PBS) twice. Cells were harvested by trypsinization and counted (Multisizer 3, Beckman Coulter, CA). BrdU incorporation assay was performed according to the manufacturer’s instructions (Roche Applied Science, Indianapolis, IN).

### Cell migration analysis

A scratch test was performed to evaluate the effect of physiological stretch on VSMCs migration. Cells were plated directly onto silicone membranes of Flexcell 6-well plates. Before stretch stimulation, when cells had reached confluence, they were scraped with a sterile 200-μl pipette tip across the layer. The culture media were replaced with fresh complete culture media, then cells were exposed to 10% stretch or remained under static conditions for 12 h. After stretch, cells were washed with PBS, then fixed with 1% paraformaldehyde. The cell-migration distance was measured at 7 points along the wound edge by use of Photoshop CS3.

### Promoter activity assay

A 2000-bp fragment of human ACE2 promoter region (length, -1908 to +1; GenBank accession no. 13557) was subcloned into the pGL3-basic vector containing firefly luciferase (Promega) by PCR with Pfu polymerase. The pGL3-ACE2 promoter-luciferase construct and phRL-TK vector (Promega) were transiently co-transfected into HASMCs by use of lipofectamine 2000 (Invitrogen) for 24 h before stretch. After being stretched for 3 h, cell extracts were prepared and ACE2 promoter activity was measured by use of the Dual-Luciferase Reporter Assay System (Promega) and the level of firefly activity was normalized to that of Renilla luciferase.

### Electrophoretic mobility shift assay (EMSA)

Nuclear protein was extracted from HASMCs by use of a nuclear protein extraction kit (Pierce), and protein concentrations were determined by the BCA method. The sequences of double-stranded gel-shift oligonucleotides for AP-1 were: 5′-CGC TTG ATG ACT CAG CCG GAA-3′, and 3′-GCG ACC TAC TGA GTC GGC CTT-5′; for NF-κB were: 5′-AGT TGA GGG GAC TTT CCC AGG C-3′, and 3′-TCA ACT CCC CTG AAA GGG TCC G-5′. The above two probes were end-labeled with biotin. The sequences mutant probe for AP-1 were: 5′-CGC TTG ATG ACT TGG CCG GAA-3′and 3′-GCG AAC TAC TGA ACC GGC CTT-5′; the sequences mutant probe for NF-κB were: 5′-AGT TGA GGC GAC TTT CCC AGG C-3′ and 3′-TCA ACT CCG CTG AAA GGG TCC G-5′. The nuclear extracts (10 μg) were mixed with labeled oligonucleotides for AP-1 or NF-κB and other important components in a total volume of 20 μl for 20 min, then separated by 6% nondenaturing acrylamide PAGE. The detailed procedures were according to the manufacturers’ instructions (Pierce).

### RNA stability

Before application of 10% stretch over a time course of 12 h, transcription inhibitor actinomycin D (5 μg/ml) was added to the HASMC media with or without stretch. ACE2 mRNA levels were determined by quantitative RT-PCR at the indicated times. β-Actin mRNA levels were measured as the internal control. The results of ACE2 mRNA degradation are expressed as ratio of mRNA levels at each time in comparison with those at the time of initial actinomycin D treatment (0 h).

### Statistical analysis

Data are presented as means ± SEM. Comparisons between groups and pairs involved one-way ANOVA or Student’s *t* test, as appropriate. Statistical significance was defined as *P*<0.05.

## Results

### Physiological stretch promotes ACE2 expression and activity *in vitro*

**Figure 1:**
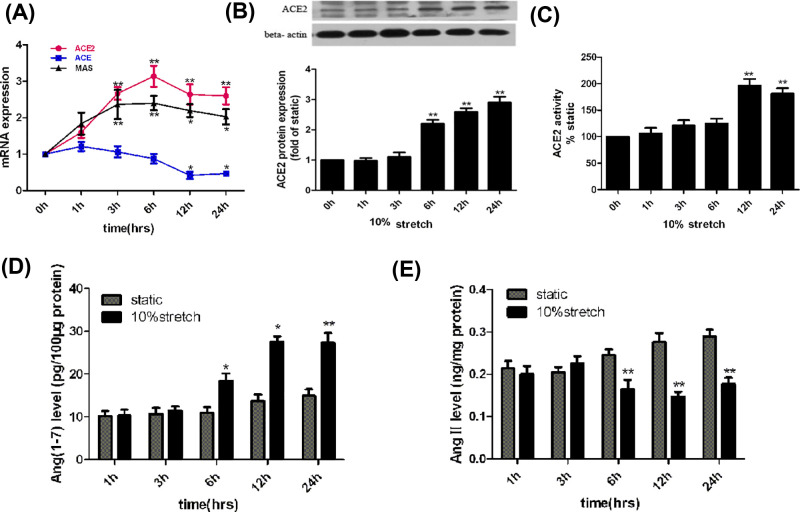
Physiological stretch regulates ACE2 expression and activity, as well as other components of RAS in HASMCs HASMCs grown on six-well Flexcell plates (10^5^/well) coated with collagen I were kept under static conditions or subjected to 10% stretch 1, 3, 6, 12, 24 h. (**A**) The mRNA levels of ACE2, ACE, MAS were determined by RT-PCR. (**B**) The ACE2 protein expression was determined by Western blot. (**C**) The enzymatic activity of ACE2 was evaluated by SELDI-TOF-MS method. (**D** and **E**) The levels of Ang-(1-7) and Ang II were determined by ELISA. Data are shown in mean ± SEM from at least three independent experiments; *P<0.05, **P<0.01 vs. static control (0 h).

We first investigated whether physiological stretch (10% elongation) affects ACE2 expression and activity in VSMCs. The cultured HASMCs were exposed to 10% stretch over a time course of 24 h. As shown in Figure 1A, stretch significantly increased ACE2 mRNA expression within 3 h, reached a maximal level at 6 h (3.1-fold), and remained elevated at 24 h in comparison with static control cells (0 h); the induction of MAS mRNA expression by stretch was in keeping with that of ACE2. In contrast, the ACE mRNA expression began to decrease after 12 h stretch stimuli, until 24 h. The ACE2 protein expression in stretched cells was also increased in a time-dependent manner, began to increase at 6 h, and remained elevated after 24 h of stretching in comparison with static control cells (Figure 1B). In addition, our results found the effect of 10% stretch on ACE2 enzyme activity was in concert with the effect of stretch on ACE2 protein expression, as shown in Figure 1C. Multiple evidence reveal that the vascular protective roles of ACE2 are closely associated with regulating the balance of Ang-(1-7) and Ang II. We also investigated the role of stretch on Ang-(1-7) and AngII level in VSMCs, as shown in Figure 1D,E; 10% stretch significantly induced Ang-(1-7) expression, but reduced Ang II level in a time-dependent manner, as compared with static control cells. These results indicated that ACE2 is a mechanical stretch sensitive gene, and physiological stretch induces a sustained elevation of ACE2 expression and activity, as well as increases Ang-(1-7) level, decreases Ang II in cultured VSMCs.

### ACE2 is implicated in regulating proliferation and migration of HASMCs mediated by physiological stretch

**Figure 2:**
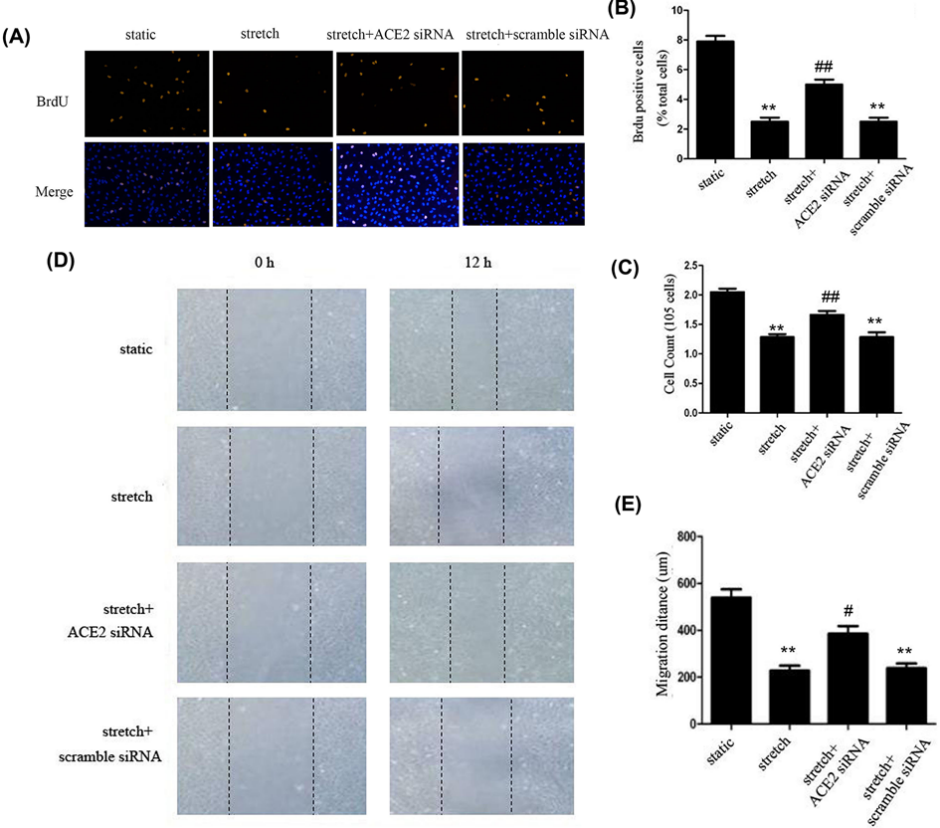
ACE2 is involved in regulating proliferation and migration of HASMCs mediated by stretch Cultured HASMCs with different conditions were exposed to 10% stretch for 12 h; cells kept under static conditions were considered as control group. (**A–C**) The proliferation of HASMCs in each group was determined by BrdU incorporation assay and cell counting method, respectively. (**D** and **E**) The cell migration was evaluated by a scratch test. Data are shown in means ± SEM from at least three independent experiments. **P<0.01 vs. static control; ^#^*P*<0.05, ^##^*P*<0.01, vs. stretch alone.

Several lines of evidence indicate that ACE2 is closely associated with vascular functions. To investigate whether ACE2 is involved in regulating VSMC functions mediated by stretch, we first investigated the effect of 10% stretch on HASMCs proliferation via BrdU incorporation assay and cell counting. As shown in Figure 2A–C, the proportion of BrdU-positive cells in stretched cells was significantly decreased in comparison with static control cells, and the results of cell counting were in concert with the Brdu incorporation assay; however, knockdown of ACE2 expression by specific siRNA, this inhibitory effect of 10% stretch on proliferation of HASMCs was significantly attenuated. In addition, we also explored the effect of ACE2 on the migration of HASMCs mediated by stretch via scratch assay, as shown in Figure 2D,E, our data showed that 10% stretch significantly suppressed the migration of HASMCs compared with static control; however, knockdown of ACE2 expression by specific siRNA partially reversed the inhibitory effect of stretch on cell migration. Thus, these findings indicate that 10% stretch-induced ACE2 is implicated in regulating the proliferation and migration of VSMCs.

### Physiological stretch increases the promotor activity of ACE2

**Figure 3:**
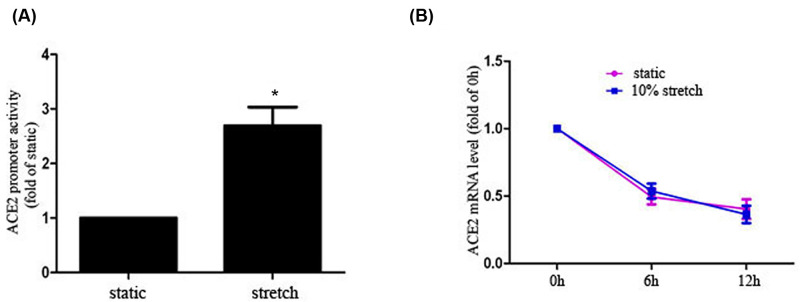
The effect of stretch on ACE2 promoter activity and mRNA stability (**A**) Cultured HASMCs were co-transfected with pGL3-ACE2 promotor-luci and phRL-TK vectors for 24 h, then exposed to 10% stretch or kept under static conditions for 3 h. The ACE2 promoter activity was determined by dual-luciferase assay and the fold changes in ACE2 promoter activity were calculated in comparison with the static control. (**B**) Cultured HASMCs were pretreated with transcription inhibitor actinomycin D (5 μg/ml) for 12 h, then cells were exposed to 10% stretch or kept under static conditions for 0, 6, 12 h; the ACE2 mRNA expression was determined by RT-PCR. Data are shown as means ± SEM from three independent experiments. *P<0.05 compared with static control.

Previous studies indicate mechanical forces could regulate genes at multiple levels, including transcriptional, post-transcriptional level. To explore the mechanism by which physiological stretch regulates ACE2 expression, we cloned a 2000-bp fragment of ACE2 promotor into the pGL3-basic-luc vector to generate pGL3-ACE2 promotor-luc, which was transiently co-transfected with phRL-TK vector into HASMCs before application of stretch. The result of dual-luciferase assay showed 10% stretch significantly enhanced ACE2 promotor activity in comparison with static conditions, as shown in Figure 3A. Besides, we also investigated the effect of 10% stretch on the mRNA stability of ACE2, as shown in Figure 3B; stretch did not affect the ACE2 mRNA stability in comparison with static conditions. These results indicate physiological stretch increases the ACE2 promotor activity, at least partially regulates ACE2 expression at the transcriptional level.

### The transcription factors, AP1 and NF-κB, are responsible for regulating ACE2 expression mediated by physiological stretch

**Figure 4:**
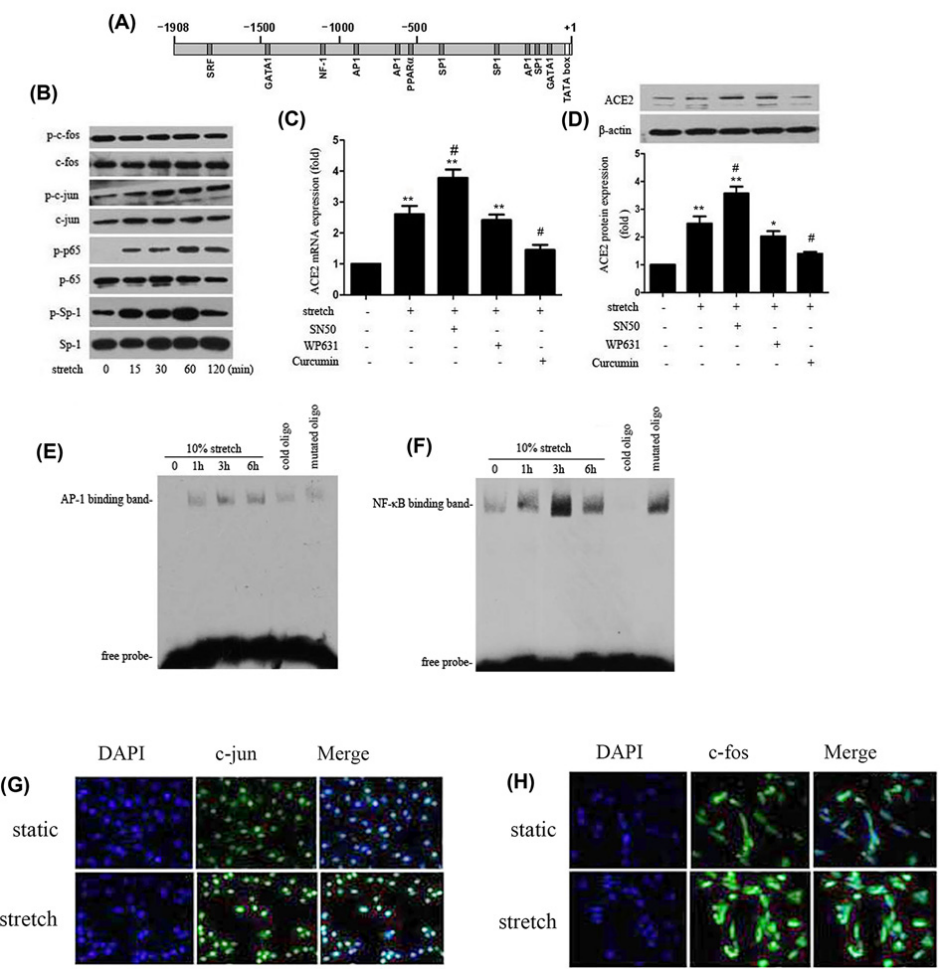
AP-1 and NF-κB were implicated in the regulation of ACE2 mediated by stretch (**A**) Potential binding sites of transcription factors in the promoter region of human ACE2 gene by TRANSFAC analysis (marked with gray boxes). (**B**) Cultured HASMCs were subject to 10% stretch for 0, 15, 30, 60, 120 min. Protein lysates were collected to detect the levels of c-jun, c-fos, p65, Sp-1, and their phosphorylated forms by Western blot. (**C** and **D**) Cultured HASMCs were incubated with inhibitors of NF-κB (SN50,18 μM), Sp-1(WP631, 100 nM), AP-1 (Curcumin, 10 μM) for 1 h, then were exposed to 10% stretch for 6 h. RT-PCR and Western blot were performed to detect ACE2 mRNA and protein expression. (**E** and **F**) Cultured HASMCs were exposed to 10% stretch for 0, 1, 3, 6 h. The nuclear protein was extracted and the DNA binding activities of AP-1 and NF-κB were determined by EMSA. (**G** and **H**) HASMCs were exposed to 10% stretch or kept under static conditions for 1 h, then cells were fixed, the expression and location of c-jun and c-fos were investigated by immunofluorescence. DAPI was used to visualize cell nuclei. Data are means ± SEM from at least three independent experiments. *P<0.05, **P<0.01 vs. static control (0 h), ^#^*P*<0.05, vs. stretch alone.

As shown in Figure 4A, the human ACE2 promotor region contains several AP-1 and Sp-1 binding sites according to TRANSFAC database analysis. Besides, a previous study by Takase et al. indicated that NF-κB was a negative regulator for ACE2 expression. First, we investigated the phosphorylation of c-fos, c-jun, p65, and Sp1 in the nucleus of cells, the WB results showed that 10% stretch induced a rapid phosphorylation of c-jun, p65, and Sp1 compared with static control (Figure 4B). Then, to determine the transcription factors mediating the regulation of ACE2 expression by stretch, we used specific inhibitors for AP-1, Sp-1, and NF-κB to block their activation before stretch stimuli, as shown in Figure 4C,D; we found that pretreatment cells with SN50 (NF-κB inhibitor) further increased ACE2 mRNA and protein expression compared with stretch alone. Curcumin (AP-1 inhibitor) obviously attenuated 10% stretch-induced ACE2 expression; however, WP631 (Sp-1 inhibitor) did not affect stretch-induced ACE2 expression, these results demonstrated that AP-1 and NF-κB were likely involved in regulating ACE2 expression mediated by stretch. Besides, we further investigated the activities of AP-1 and NF-κB, the EMSA results showed 10% stretch significantly increased the two transcription factors’ activities compared with static conditions (Figure 4E,F). In addition, we also investigated the expression of c-jun and c-fos by immunofluorescence, as shown in Figure 4G,H; 10% stretch significantly increased the c-jun expression mainly in nucleus region compared with static conditions. However, the increased c-fos expression induced by stretch was mainly in cytoplasm, not in nucleus of cells. These above results demonstrated that transcription factors, AP-1 and NF-κB, were involved in regulating ACE2 expression by physiological stretch.

### PKCβII and JNK1/2 pathways are involved in stretch-induced ACE2 expression

**Figure 5:**
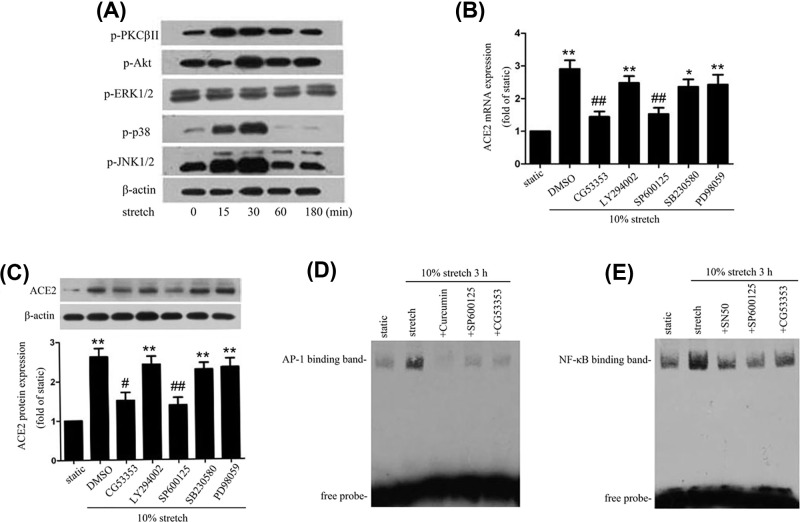
PKCβII and JNK1/2 signaling pathways were involved in stretch-induced ACE2 expression (**A**) Effect of stretch on activations of mitogen-activated protein kinase (MAPK), Akt, and PKCβII pathways. Cultured HASMCs were subjected to 10% stretch for 0, 15, 30, 60, 180 min. The phosphorylation of ERK1/2, p38, JNK1/2, Akt and PKCβII were determined by Western blot. (**B** and **C**) Cultured HASMCs were pretreated with PKCβII inhibitor (CG53353, 100 nM), Akt inhibitor (LY294002, 50 μM), p38 inhibitor (SB203580, 10 μM), JNK1/2 inhibitor (SP600125, 20 μM), ERK1/2 inhibitor (PD98059, 30 μM) for 1 h respectively. DMSO was used as a negative control, then were exposed to 10% stretch for 6 h. RT-PCR and Western blot were performed to detect ACE2 mRNA and protein expression. (**D** and **E**) The effect of blockade of PKCβII and JNK1/2 pathways on AP-1 and NF-κB DNA binding activities in stretched cells. HASMCs were incubated with AP-1 inhibitor (Curcumin, 10 μM), NF-κB inhibitor (SN50, 18 μM), PKCβII inhibitor (CG53353, 100 nM), and JNK1/2 inhibitor (SP600125, 20 μM) for 1 h, respectively, then exposed to 10% stretch for 3 h. Cells kept under static conditions were considered as static control cells. The nuclear protein was extracted to detect the AP-1 and NF-κB DNA binding activities by EMSA. Data are means ± SEM from at least three independent experiments. *P<0.05, **P<0.01 vs. static control (0 h), ^#^*P*<0.05, ^##^*P*<0.01vs. DMSO.

To determine the pathways involved in stretch-induced ACE2 expression, we investigated the effect of 10% stretch on activation of MAPK, Akt, and PKC signaling pathways. As shown in Figure 5A, 10% stretch induced a transient phosphorylation of p38, JNK1/2, Akt, and PKCβII in comparison with static conditions (0 h). To identify the key signaling pathways mediating the regulation of ACE2 expression by stretch, we added specific inhibitors of p38, JNK1/2, ERK1/2, PKCβII, and Akt to the media of cultured HASMCs 1 h before application of stretch for 6 h. ACE2 expression in each group was determined by Western blot and RT-PCR, the experimental results showed that inhibitors of PKC βII(CG53353) and JNK1/2 (SP600125) significantly attenuated stretch-induced ACE2 expression (Figure 5B,C). However, the inhibitors of Akt (LY294002), p38 (SB203580), and ERK1/2 (PD98059) had no effect on stretch-induced ACE2 expression. To further examine whether JNK1/2 and PKCβII pathways affect the DNA binding activities of AP-1 and NF-κB. HASMCs were pretreated with CG53353 (PKCβII inhibitor) and SP600125 (JNK1/2 inhibitor) 1 h before stretch treatment, then subjected to 10% stretch for 3 h, and the EMSA results revealed that both CG53353 and SP600125 could attenuate stretch-induced DNA binding activities of AP-1 and NF-κB (Figure 5D,E), so the activations of PKCβII and JNK1/2 pathways are required for the increased activities of AP-1 and NF-κB induced by stretch. These results indicated the physiological stretch-induced activations of PKCβII and JNK1/2 pathways are responsible for regulating of ACE2 expression.

## Discussion

In the present study, we demonstrate for the first time that (i) physiological stretch increases the expression and activity of ACE2 in VSMCs, which is involved in stretch mediated proliferation and migration of cells and (ii) the activation of JNK1/2 and PKCβII as well as transcription factors AP-1 and NF-κB may contribute to the up-regulation of ACE2 induced by physiological stretch.

The blood vessels are constantly exposed to biomechanical forces in the form of cyclic stretch and shear stress, and the VSMCs in the media of the vessel are mainly affected by stretch. Accumulating evidence indicate physiological stretch with the magnitude of 5–10% are crucial for the normal vascular homeostasis, with characteristics of reduced proliferation and migration, maintaining the contractile phenotype of SMCs [3,15]. However, the pathologically increased stretch as occurred during hypertension are closely associated with increased proliferation, migration, dedifferentiation of VSMCs, contributing to the adverse vascular remodeling [16–18].

The RAS plays important roles in regulating vascular functions and blood pressure, and the overactivation of RAS is the key pathological mechanism for some vascular diseases, including hypertension and atherosclerosis. Several previous reports indicate that some major components of RAS are implicated in stretch mediated cellular functions. Chiu et al. demonstrated that 20% stretch increased Ang II expression in rat VSMCs [13]; evidence from Wang et al. found stretch increased the secretion of Ang II in cultured VSMCs [19]; Liu et al. revealed that stretch significantly up-regulated the protein expression of Ang II type1 receptor (AT_1_R) in SHR VSMCs, contributing to the cell proliferation [14]; indeed, growing evidence suggest AT_1_R is an important mechano-sensor in vascular cell membrane [20,21]. In our previous work, we found mechanical stretch increased ACE expression via inhibition of microRNA-145, promoting the VSMCs phenotypic modulation [22]. These above experimental results indicate that RAS plays important roles in regulating functions of vascular cells by stretch; however, it is not clear whether ACE2 is also involved in this process. In a recent study by our group, we found shear stress can regulate ACE2 expression, contributing to maintain normal endothelial cell functions [23]. In the present study, we investigated the effect of physiological stretch on ACE2 expression and activity *in vitro.* Our data showed 10% stretch significantly increased the expression and activity of ACE2, as well as the MAS mRNA expression, but decreased the ACE expression, suggesting that ACE2 is also sensitive to stretch treatment. ACE2 is considered as an endogenous negative regulator of RAS, exhibiting cardiovascular protective roles mainly via catalyzing Ang II into Ang-(1-7). In the present study, we found 10% stretch induced a time-dependent elevation of Ang-(1-7) level. In contrast, the Ang II level was obviously decreased in stretched cells. Despite ACE and other enzymes are also responsible for AngII and Ang-(1-7) generation, but our results suggest that the levels of the two active peptides induced by physiological stretch at least partially due to up-regulation of ACE2.

In vascular vessels, ACE2 is mainly expressed in ECs and SMCs. Numerous studies suggest ACE2 is an important regulator for normal functions of VSMCs. Sahara et al. reported that deletion of ACE2 promoted the proliferation of VSMCs, accompanied with increased Ang II level and pro-inflammatory genes [24]. Song et al. revealed recombinant ACE2 suppressed Ang II-induced oxidative stress and VSMCs proliferation [25]. Zhang et al. revealed that Ad-ACE2-transfected VSMCs showed a significant reduction of proliferation and migration [26]. Thus, these experimental data indicate ACE2 markedly inhibited VSMCs proliferation and migration. It is well known that physiological stretch is a major determinant for maintaining VSMCs functions; however, whether ACE2 is implicated in regulating VSMCs functions under stretch treatment is not clear. In the present study, we found 10% stretch significantly reduced the proliferation and migration of HASMCs, which was consistent with other previous studies. Furthermore, we used specific siRNA to inhibit the stretch-induced ACE2 expression. Our results showed that the inhibitory effects of stretch on VSMCs proliferation and migration were markedly attenuated as compared with control siRNA. Thus, our results indicated that ACE2 is involved in regulating VSMCs proliferation and migration mediated by physiological stretch.

Despite growing evidence have proved the vascular protective roles of ACE2, making it a potential therapeutic target for many vascular diseases; however, the regulatory mechanisms of ACE2 expression is less known as compared with its biological roles. Several recent studies explored the regulatory mechanisms of ACE2 expression, indicating ACE2 can be modulated at different levels. Evidence from Zhang et al. revealed transcription factor C/EBPβ can interact with ACE2 promoter to induce its expression in high glucose treated cardiomyocytes [27]. Turner discovered that ACE2 is subject to post-transcriptional regulation by miR-421 in cardiac myofibroblasts [28]. Moran et al. reported resveratrol increases ACE2 expression in HASMCs in a sirtuin1-dependent manner [29]. Indeed, there are complex interactions between the ACE/AngII/AT1R axis and ACE2/Ang-(1-7)/MAS axis. Zhu et al. demonstrated that activation of angiotensin II type 2 receptor increases ACE2 expression and activity in ECs, contributing to the anti-inflammatory effect [30]. In Ang II-mediated hypertension mice, the expression and activity of ACE2 significantly decreased via Ang II-mediated ACE2 internalization and degradation [31]. Mechanical forces can regulate gene expression at different levels, including transcriptional and post-transcriptional, even a mechano-sensitive gene could be modulated at multiple levels, such as eNOS. Previous studies revealed that laminar shear stress not only enhanced the promoter activity of eNOS, but also increased its mRNA stability [32,33]. To elucidate the mechanism by which stretch regulate ACE2 expression, we first explored the effect of stretch on ACE2 promoter activity as well as its mRNA stability. Our results showed stretch increased the promoter activity of ACE2, but did not affect its mRNA stability, suggesting stretch modulate ACE2 expression mainly at transcriptional level. The molecular mechanisms underlying the stretch regulates VSMCs functions are not fully clear, but multiple evidence indicate several transcription factors (e.g. AP-1, Sp-1, NF-κB) and signaling pathways (e.g. MAPK, PKC, Akt) are involved in the mechano-transduction process. By TRANSFAC analysis, we found the promoter of ACE2 contains several putative binding sites for AP-1 and Sp-1. Besides, the protective roles of ACE2 were reported to be associated with decreased NF-κB activity [34,35]. In the present study, our results found the nuclear levels of p-c-jun, p-p65 and p-Sp1, as well as the activities of AP-1 and NF-κB were significantly increased in stretched cells, then, by use of specific inhibitors, we found inhibition of AP-1 significantly attenuated stretch-induced ACE2 expression; however, inhibition of NF-κB further increased ACE2 expression and inhibition of Sp1 did not affect ACE2 expression. These results indicated AP-1 and NF-κB are likely involved in regulating ACE2 expression by stretch, but NF-κB maybe a negative regulator for ACE2 expression. It is worth to note that there is no putative binding site for NF-κB in ACE2 promotor, thus, we speculate that NF-κB influence ACE2 expression in an indirect way. In addition, the direct interaction between AP-1 and ACE2 promoter has not been studied here, which warrants further investigation. In addition, c-jun and c-fos are two main components of AP-1. Our immunofluorescence results showed that stretch markedly increased c-jun expression in the nucleus of cells, while c-fos mainly located in the cytoplasm, indicating that c-jun may play a critical role in regulating ACE2 expression by stretch.

Numerous studies have proved some intracellular pathways are involved in stretch signal transduction, including PKCs, PI3K/Akt, and MAPKs. Activation of these pathways by stretch drives patterns of gene expression to modulate VSMCs functions. PKCs are a family of Ser/Thr kinases that play an important role in stretch-mediated VSMC migration [36]. PKCβII, which is a conventional PKCs isoform, was found to be involved in high glucose-induced inhibition of ACE2 expression in rat VSMCs [37]. The MAPKs are also a family of Ser/Thr kinases that consist of three pathways including ERK1/2, JNK1/2, and p38. Differential patterns of stretch can activate different molecules of MAPKs, leading to the activation of their downstream transcription factors, such as AP-1 and NF-κB [38,39]. Ang II is the main substrate of ACE2, but excess Ang II can suppress the ACE2 expression via AT1R-ERK/p38 pathway [40]. The PI3K/Akt pathway is also involved in stretch mediated several VSMC functions, especially proliferation [41]. In the present study, stretch induced a rapid activation of the PKCβII, Akt, and MAPKs pathways, as shown in Figure 5A, which was consistent with previous studies. In order to identify the pathways contributing to stretch-induced ACE2 expression, we used specific inhibitors to block the activation of these pathways. As a result, both CG53353 (PKCβII inhibitor) and SP600125(JNK1/2 inhibitor) significantly attenuated stretch-induced ACE2 expression. However, other inhibitors specific for PI3K/Akt, p38 and ERK1/2 had no effect on stretch-induced ACE2 expression. Besides, our data also demonstrated that blockade of PKCβII and JNK1/2 pathways obviously decreased AP-1 and NF-κB activities in stretched VSMCs, indicating that PKCβII and JNK1/2 are the important upstream signaling molecules of transcription factors, AP-1 and NF-κB. Collectively, these results suggest that PKCβII and JNK1/2 pathways are implicated in stretch-induced ACE2 expression.

## Limitation

We demonstrate *in vitro* that physiological stretch significantly increases ACE2 expression and activity, which is involved in reducing proliferation and migration of stretched VSMCs. However, the role of ACE2 in stretch mediated VSMCs functions has not been investigated *in vivo*, especially in human aortic tissues from normal people and hypertensive patients, which warrant further investigation. Nevertheless, the limitation does not negate the evidence that ACE2 plays an important role in stretch mediated proliferation and migration of VSMCs.

**Figure 6:**
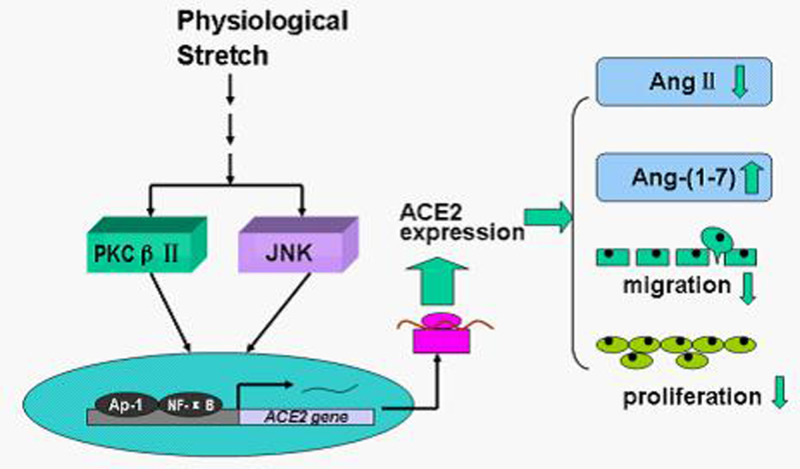
Scheme of the roles of ACE2 in physiological stretch mediated cellular functions of HASMCs and the underlying molecular mechanism

In summary, as shown in Figure 6, our study demonstrates that ACE2 is up-regulated by physiological stretch, contributing to inhibit VSMCs proliferation and migration. The mechanisms underlying by which stretch regulates ACE2 expression may involve the activation of PKCβII and JNK1/2 pathway, as well as transcription factors, AP-1 and NF-κB. Thus, in the present study, we further demonstrate that ACE2 is also sensitive to mechanical stretch, and plays important roles in regulating cellular functions. Such findings may provide insights to further understand the interactions between RAS and mechanical forces.

## Abbreviations

ACE: angiotensin-converting enzyme
ACE2: angiotensin-converting enzyme 2
Ang II: Angiotensin II
Ang-(1-7): Angiotensin-(1-7)
AP-1: activator protein-1
EMSA: electrophoretic mobility shift assay
HASMCs: human vascular smooth muscle cells
MAPKs: mitogen-activated protein kinases
NF-κ B: nuclear factor kappa B
PKC: protein kinases C
RAS: renin–angiotensin system
Sp1: specificity protein 1
